# Mantle Cell Lymphoma With Non-traumatic Splenic Rupture Requiring Emergency Splenectomy

**DOI:** 10.7759/cureus.24675

**Published:** 2022-05-02

**Authors:** Patrick D Plummer, Benjamin Yglesias, Adam Swiger, Penelope Mashburn

**Affiliations:** 1 Department of Surgery, Trumbull Regional Medical Center, Warren, USA

**Keywords:** non-hodgkin’s lymphomas, spontaneous rupture, splenectomy, splenic rupture, mantle cell lymphoma

## Abstract

Mantle cell lymphoma (MCL) is a type of non-Hodgkin (B-cell) lymphoma (NHL) with manifestations ranging from indolent to aggressive disease. This type of NHL is predominately found in western countries and affects men more often than women (M:F 2:1). The median age of diagnosis with the disease is around 60 years of age. In this report, the patient is a 68-year-old female who had an atraumatic splenic rupture with no past medical history of trauma. She presented to the emergency department with severe abdominal pain in her left upper quadrant. An emergency splenectomy was executed successfully, and the patient was stabilized. In this case report, we will discuss the pathogenesis, clinical presentation, known clinical treatment, diagnostic testing, and atraumatic splenic rupture.

## Introduction

Mantle cell lymphoma (MCL) is a type of non-Hodgkin (B-cell) lymphoma (NHL) that can involve the lymph nodes, spleen, blood, and bone marrow [1-4]. In 75% of cases, lymph nodes are the primary site of neoplasm [1,-4]. Diffuse and localized lymph node involvement are present in 57% and 30% of cases, respectively [1,2,3,4]. Extra-nodal sites include bone marrow (69%-79%), spleen (47%), peripheral blood (36%), gastrointestinal tract (18%), colon (13%), liver (13%), head and neck (12%) [2,4]. MCL comprises about 3% to 10% of adult-onset NHL [4]. This type of NHL is known to be highly aggressive with a short remission period to standard therapies and an increased risk of relapse. The two major subgroups of MCL are divided into Nodal and Leukemic non-nodal MCL. Nodal MCL is the most common and aggressive type of MCL. This subtype usually presents with overexpression of SOX11 and a high degree of genomic instability [4]. Leukemic non-nodal MCL commonly presents with leukocytosis and splenomegaly in 10%-20% of patients [4]. The expression of SOX11 is negative and patients have higher genome stability.

In the case of splenomegaly, surgical removal or transcatheter ablation of splenic parenchyma is a vital option [5]. Patients with a splenic rupture showing clinical signs of hypotension, diaphoresis, pain in the left upper quadrant (LUQ), and confusion should be evaluated immediately [5]. Pre-operational management of patients with splenic rupture is to achieve hemodynamic stability [5]. Blood transfusions and vasopressors can help stabilize the patient until surgical procedures can be performed.

Patients with MCL can present with various signs and symptoms. Newly diagnosed patients usually present with lymphocytosis detected by peripheral blood flow cytometry with severe cytopenia. Roughly 14%-25% of patients diagnosed with MCL present with fever, malaise, weight loss, and night sweats [4]. The patient can also present with hepatosplenomegaly [4]. Most patients will present with an elevated white blood cell (WBC) count, low red blood cells (RBC) and platelets. Depending on the location of the malignancy, patients can have lymphadenopathy in multiple affected nodes.

Immunophenotypically, MCL B-cells markers are CD5, CD19, CD20, CD22, PAX5, CD79a and Cyclin D1 [1,2,4]. MCL also expresses cell surface immunoglobulins IgM and IgD [4]. The overexpression of SOX11 has been recognized as a specific marker for diagnosis of MCL [3]. Although there is no standard care or treatment for MCL, the management of patients with MCL is based on the patient's age and fitness. Therapies include incorporating rituximab and cytarabine, with acute stem cell transplantation (ASCT) if eligible [4]. Young fit patients are managed with ASCT with combination therapy of rituximab, while older patients are managed with rituximab only [6]. Complications of MCL include anemia, thrombocytopenia, and neutropenia. In rare cases, patient can present with splenomegaly with an increase of risk spontaneous splenic rupture. In this case report, we describe a patient with newly diagnosed MCL who presented to the emergency room with a spontaneous splenic rupture requiring emergent surgical intervention.

## Case presentation

The patient is a 68-year-old female with a past medical history of hypothyroidism and newly diagnosed MCL. The patient presented to the emergency department (ED) complaining of sharp abdominal pain located in the LUQ and overall weakness. The patient started feeling progressively weak with abdominal pain four days prior. The patient was afebrile and hypotensive. Table 1 shows the patient's lab values prior to the surgery procedure.

**Table 1:** The patient's hematology lab values taken during her hospitalization prior to surgery.

Test	Patient Labs	Reference Range	Units
Total white blood cell count (CBC)	55.3	4 - 10.4	10^3^/uL
Lymphocytes	48	20 -40	%
Monocytes	5.96	2.1 - 8.1	%
Neutrophils	33.9	40 - 60	%
Platelets	245	165 - 415	10^3^/uL
Hemoglobin	7.7	13.3 - 16.2	g/dL
Hematocrit	25.1	38.8 - 46.4	%
Mean Corpuscular Volume (MCV)	102.4	80 - 100	fL

**Figure 1:**
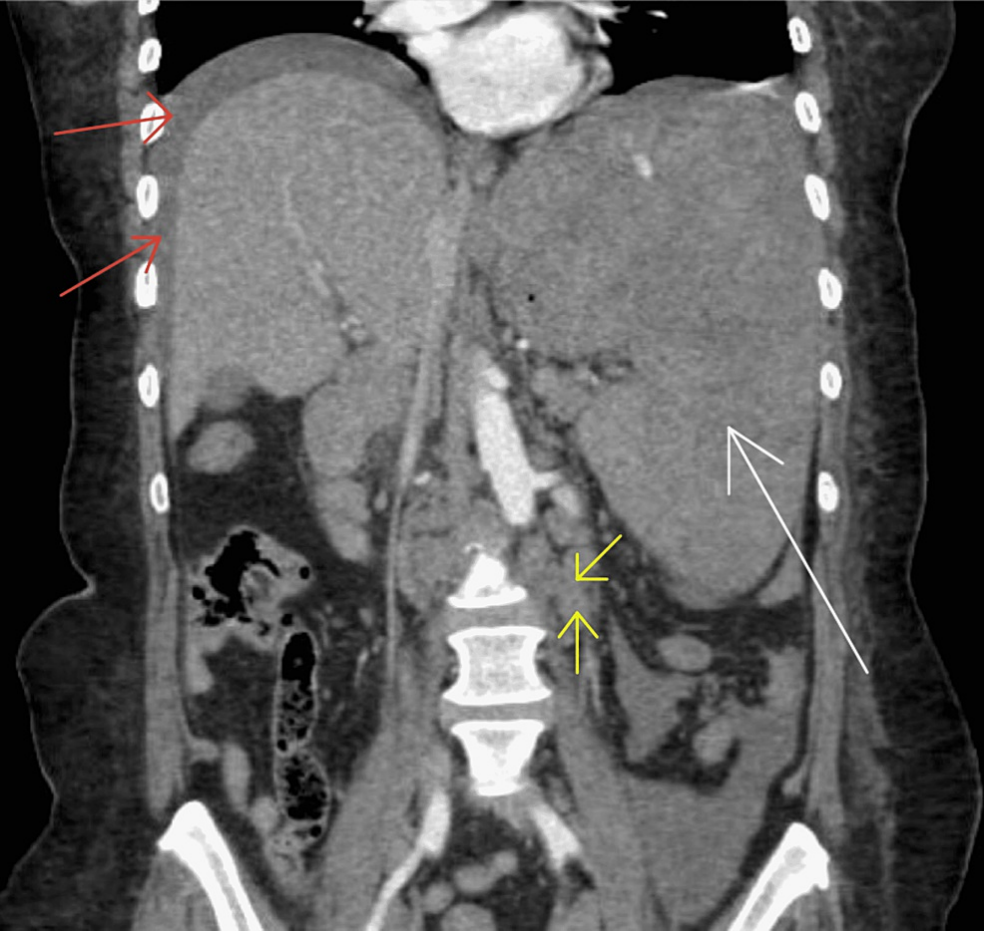
Coronal computerized tomography (CT) scan. Red arrows show gross hemoperitoneum. White arrow shows splenomegaly. Yellow arrows show enlarged abdominal lymph nodes.

**Figure 2:**
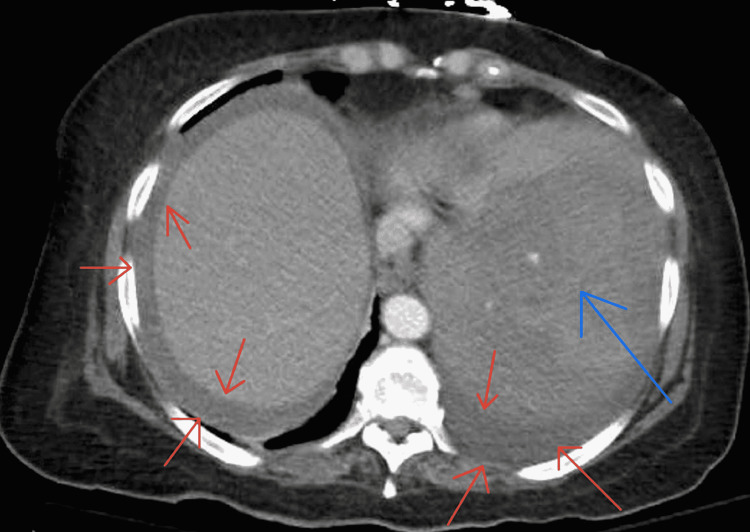
Axial computerized tomography (CT) scan. Red arrows show gross hemoperitoneum. Blue arrow shows splenomegaly.

On the day of her admission, the patient was found lying on the floor with severe pain in her LUQ. The patient reiterated that she did not fall, but rather voluntarily lay on the floor to alleviate her pain. The patient denied any loss of consciousness or traumatic falls. The patient also stated that she does not have a past medical history of traumatic events. Physical examination of the patient’s abdomen reveals tenderness to palpation of the LUQ and left lower quadrant (LLQ). The patient showed no guarding or rebound tenderness. Once the patient was partially stabilized, she was taken for an anterior/posterior computerized tomography (CT) of her abdomen (Figures 1, 2). The results of the CT showed enlarged spleen with rupture, diffuse hemoperitoneum, and enlarged lower thoracic and abdominal lymph nodes consistent with her MCL (Figures 1, 2). Given the patient’s unstable hemodynamics, with a blood pressure (BP) 85/64, the patient was scheduled for an emergency splenectomy.

The patient was transferred emergently to the operating room (OR) and placed in the supine position on the operating table. A midline laparotomy was performed. Upon entry into the peritoneum, gross hemoperitoneum was noted. Over 1 liter of coagulated blood was removed from the abdomen by hand. There was gross bleeding from the posterior lateral surface of the spleen due to a laceration. The splenic arteries were located and clamped. The spleen was removed and sent to pathology. Patient was transferred to the intensive care unit (ICU) for further monitoring. The patient was given two units of RBC during the operation and another two units post-operational (post-op). Once the patient’s BP stabilized, previously administered vasopressors were discontinued, and she remained in the ICU for further monitoring. She had a stable recovery and was eventually discharged to rehabilitation facility. She underwent one round of chemotherapy, but declined any further intervention. The patient did not receive post-splenectomy vaccinations against Streptococcus pneumonia, Hemophilus influenza, and Neisseria meningitidis.

**Figure 3:**
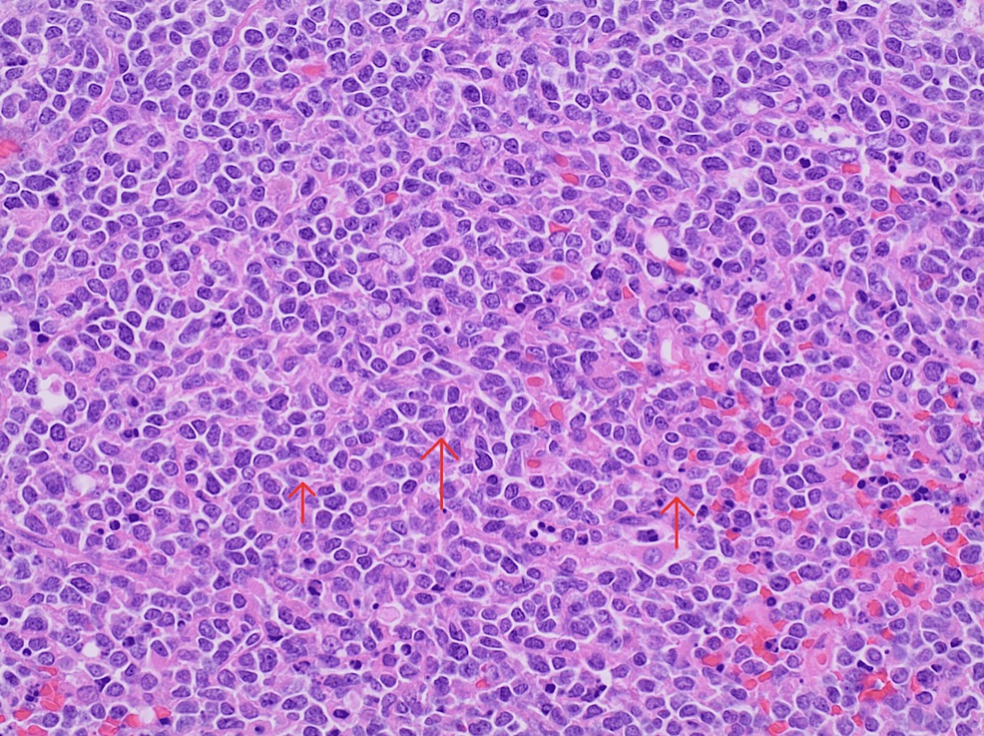
Mantle cell lymphoma (MCL) histology slide of pleomorphic cells. Red arrows show irregular nuclear contour, relatively condensed chromatin, and enlarged cytoplasm in MCL cells.

**Figure 4:**
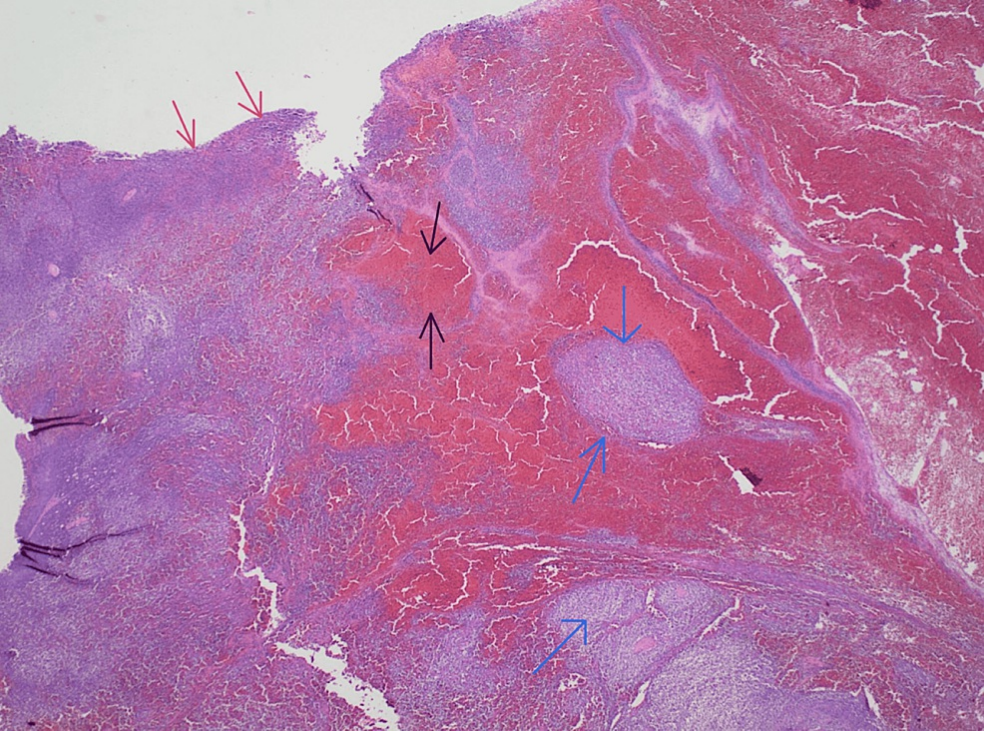
Histology slide of splenic capsule (red arrows) with underlying parenchyma (black arrows). Mantle cell pattern is shown; the neoplasm surrounds reactive germinal centers (blue arrows).

The pathology report on the excised spleen revealed a grossly enlarged, roughly oval-shaped spleen with overall dimensions of approximately 27.8 x 20.5 x thickness ranging from 3.0 to 8.2 cm. The total weight was 2,350 g. The capsule had a smooth texture. There was a V-shaped laceration with an approximate length, weight, and depth of 18.5 cm x 5.2 cm x 4.2 cm, respectively. There were multiple sub-capsular and parenchymal hemorrhages along with wedge-shaped infarctions. The parenchymal hemorrhage associated with the lacerations extended approximately 18 cm. Histology shows pleomorphic cells with irregular nuclear contour, relatively condensed chromatin, and enlarged cytoplasm (Figure 3). The underlying parenchyma shows proliferating germinal center with in the mantle zone of the spleen (Figure 4). 

## Discussion

While spontaneous splenic rupture in patients with MCL is rare, this unusual complication should be considered in patients presenting with abdominal pain. In MCL, an enlarged spleen accounts for nearly 40% of cases [6,7]. In aggressive forms of MCL, highly proliferative neoplasm can increase tensile forces from within the spleen [6,7]. It has been theorized, that the rate of splenic expansion outpaces the rate of splenic capsule compensation, which creates high sheering tensile forces of the splenic tissue leading to rupture [7]. Another potential possibility leading to splenic rupture is compression of abdominal musculature on splenic tissue during physical activities [7]. The risk of splenic rupture increases drastically with age. This is mostly due to the changes in the behaviors of hematologic cell types and anatomical abnormalities leading to splenic vulnerability [6,7].

The clinical presentation of MCL varies from patient to patient; however, some clinical signs do correlate. The most common clinical feature of splenic ruptures is abdominal pain, which has been reported to be present in nearly 70% of cases [6,7]. Nevertheless, the absence of abdominal pain does not rule out splenic suspicions in patient with MCL. According to a study by Gorg et al., 41 patients that were diagnosed with MCL and had an associated splenic rupture were reviewed [8]. Associated abdominal pain was established in nearly 60% of patients prior to diagnosis [8]. Signs of hemodynamic instability like hypotension and tachycardia were also signs of possible splenic rupture in patient with MCL. Kehr’s sign, which is defined as left hypochondriac pain radiating to the left shoulder, occurred in nearly 20% of patients with splenic rupture [7].

The diagnosis of spontaneous splenic rupture associated with MCL relies on both clinical and confirmatory imaging studies [6,7]. The sensitivity of ultrasound for the detection of splenic rupture ranges from 72% to 78% with a specificity of 91% to 100%, making ultrasonography an essential tool for screening splenic ruptures [6,7]. Other diagnostic testing includes computerized tomography (CT), positron emission tomography (PET), and diagnostic peritoneal lavage [6,7].

In this case, it can be argued that the approach of management of this patient being newly diagnosed with MCL three weeks prior, should have been to undergo an ultrasound screening for splenomegaly [6,7]. Given the cost-effectiveness of this diagnostic tool, quick identification could have been useful to further investigate the possibility of splenomegaly. It could be suggested that patients diagnosed with MCL and experiencing symptoms of LUQ pain, early satiety, weakness, and fatigue should be evaluated for prophylactic splenectomy [6,7]. Prophylactic splenectomy could be effective in managing high-risk patients with MCL to prevent fatal complications.

## Conclusions

The awareness of spontaneous splenic rupture in patients with MCL is rare but should be considered a possible complication. Uncontrollable progression through the cell cycle can present with a highly aggressive neoplasm that can metastasize to various lymphoid tissues, which serve as a reservoir for the immune system. In high-risk patients, presenting with LUQ pain and hypotension, the probability of splenic rupture can be considered a differential in patients with MCL. Understanding the structural and physiologic consequences of an enlarging spleen within the abdominal cavity is important for informational screening and treatment decisions. Using cost-effective methods like ultrasonography can be used to screen patients with MCL for possible splenomegaly. An early diagnosis of splenomegaly can help determine if a prophylactic splenectomy is needed. In patients with MCL, the early identification of splenomegaly is vital for the patient's safety and survival. While this type of NHL has a low survival rate and an increased risk of relapse, the usage of simple diagnostic tools can help prevent further complications associated with atraumatic splenic rupture.

## References

[1] Vose JM (2017). Mantle cell lymphoma: 2017 update on diagnosis, risk-stratification, and clinical management. Am J Hematol.

[2] Li S, Xu J, You MJ (2021). The pathologic diagnosis of mantle cell lymphoma. Histol Histopathol.

[3] Sander B, Quintanilla-Martinez L, Ott G (2016). Mantle cell lymphoma--a spectrum from indolent to aggressive disease. Virchows Arch.

[4] Qie S, Diehl JA (2016). Cyclin D1, cancer progression, and opportunities in cancer treatment. J Mol Med (Berl).

[5] Ye H, Desai A, Zeng D, Romaguera J, Wang ML (2018). Frontline treatment for older patients with mantle cell lymphoma. Oncologist.

[6] Eyerer F, Gardner JA, Devitt KA (2021). Mantle cell lymphoma presenting with lethal atraumatic splenic rupture. Autops Case Rep.

[7] Tan CB, Rajan D, Majeed S, Ahmed S, Freedman L, Mustacchia P (2012). Pathologic rupture of the spleen in mantle-cell-type non-Hodgkin's lymphoma. Case Rep Med.

[8] Görg C, Cölle J, Görg K, Prinz H, Zugmaier G (2003). Spontaneous rupture of the spleen: ultrasound patterns, diagnosis and follow-up. Br J Radiol.

